# Investigation of Fugitive Aerosols Released into the Environment during High-Flow Therapy

**DOI:** 10.3390/pharmaceutics11060254

**Published:** 2019-06-01

**Authors:** James A. McGrath, Ciarraí O’Toole, Gavin Bennett, Mary Joyce, Miriam A. Byrne, Ronan MacLoughlin

**Affiliations:** 1 School of Physics & Centre for Climate and Air Pollution Studies, Ryan Institute, National University of Ireland Galway, H91 CF50 Galway, Ireland; C.OTOOLE9@nuigalway.ie (C.O.); miriam.byrne@nuigalway.ie (M.A.B.); 2 Aerogen, IDA Business Park, Dangan, H91 HE94 Galway, Ireland; GBennett@aerogen.com (G.B.); MJoyce@aerogen.com (M.J.); RMacLoughlin@aerogen.com (R.M.)

**Keywords:** nebuliser, exhaled aerosol, fugitive emissions, secondary exposure, aerosol, inhalation therapy

## Abstract

Background: Nebulised medical aerosols are designed to deliver drugs to the lungs to aid in the treatment of respiratory diseases. However, an unintended consequence is the potential for fugitive emissions during patient treatment, which may pose a risk factor in both clinical and homecare settings. Methods: The current study examined the potential for fugitive emissions, using albuterol sulphate as a tracer aerosol during high-flow therapy. A nasal cannula was connected to a head model or alternatively, a interface was connected to a tracheostomy tube in combination with a simulated adult and paediatric breathing profile. Two aerodynamic particle sizers (APS) recorded time-series aerosol concentrations and size distributions at two different distances relative to the simulated patient. Results: The results showed that the quantity and characteristics of the fugitive emissions were influenced by the interface type, patient type and supplemental gas-flow rate. There was a trend in the adult scenarios; as the flow rate increased, the fugitive emissions and the mass median aerodynamic diameter (MMAD) of the aerosol both decreased. The fugitive emissions were comparable when using the adult breathing profiles for the nasal cannula and tracheostomy interfaces; however, there was a noticeable distinction between the two interfaces when compared for the paediatric breathing profiles. The highest recorded aerosol concentration was 0.370 ± 0.046 mg m^−3^ from the tracheostomy interface during simulated paediatric breathing with a gas-flow rate of 20 L/min. The averaged MMAD across all combinations ranged from 1.248 to 1.793 µm by the APS at a distance of 0.8 m away from the patient interface. Conclusions: Overall, the results highlight the potential for secondary inhalation of fugitive emissions released during simulated aerosol treatment with concurrent high-flow therapy. The findings will help in developing policy and best practice for risk mitigation from fugitive emissions.

## 1. Introduction

Aerosol therapy is recognised as an effective method of administrating pharmaceuticals. The procedure involves generating an aerosol for drug delivery to the lungs to aid in the treatment of respiratory diseases. Nebulised medical aerosol has a typical particle size distribution of 1 to 5 μm [1]. If such aerosol particles escape into the indoor environment, they may remain airborne for periods of several hours [2,3,4].

Aerosol generation technologies deliver drugs to the lungs of neonatal, paediatric and adult patients through a variety of patient interfaces. Several factors influence aerosol drug delivery to the lungs, including the aerosol-generating device, the size distribution of the inhaled aerosol, the gas-flow rate, the inhalation pattern and the type of interface [5,6,7,8,9].

Dysart et al. [10] reported that the use of high-flow nasal therapy (HFNT) devices is rapidly growing in the clinical setting, for different age groups with a range of disease conditions. High-flow nasal therapy studies have reported gas-flow rates of 3 to 20 L/min for paediatrics, and 5 to 60 L/min for adults [11,12,13,14]. Studies have reported that several factors influence the quantity of aerosol available for inhalation, including the rate of gas flow delivered, the size of the nasal prongs, humidification, the size of the aerosol droplets and the aerosol generator type and position [11,13,15]. It is likely that these factors also influence the quantity of aerosol available to be released as fugitive emissions.

Fugitive emissions consist of aerosol that has been exhaled from the patient and/or aerosol that escaped from the system prior to inhalation. Caregivers and family members are susceptible to unintended inhalation of medication. In the occupational setting, studies have highlighted concerns regarding secondary exposure to aerosolised sustained release lipid inhalation targeting (SLIT) cisplatin, pentamidine, adeno-associated serotype 2 vector containing cystic fibrosis transmembrane conductance regulator complementary DNA (tgAAVCF) and ribavirin [16,17,18,19]. Additionally, after entering the profession, respiratory therapists are reported to have an increased risk of developing asthma, which, in part, may be due to exposure to a range of aerosolised substances in the clinical setting [20,21].

To date, only a limited number of studies have examined the potential for fugitive releases during aerosol therapy. Using photographic imaging, Somogyi et al. [22] captured the release of saline aerosol that originated in a patient’s lung, even when the patient was wearing a facemask. In addition, high-definition videos have been used to estimate the dispersion distance of smoke particles, which mimicked exhaled aerosol released from non-invasive ventilation and mask ventilation [23,24]. Ari et al. [25] examined the mechanical ventilation of a patient with and without expiratory filters, and in the cases without use of a filter, a 160-fold increase of the deposited drug was observed on the exhaust port. It was estimated that greater than 45% of the nominal dose could be fugitively released. Saeed et al. [26] examined exhalation filters when using jet and vibrating mesh nebulisers (VMN) and found that up to 50% of the generated aerosol was released from the system, as fugitive aerosol, into the surrounding environment.

Elmashae et al. [27] measured aerosol concentrations, released from an adult facemask, in the breathing zone of a manikin, simulating a home-attending healthcare worker at three different distances. Total aerosol mass concentration ranged from 0.002 to 0.010 mg/m^3^ depending on the distance of the manikin relative to the aerosol source, although the medication type was not deemed to be an influencing factor. McGrath, et al. [28] quantified time-series aerosol concentrations and the size distribution of fugitive aerosol emissions from facemask and mouthpiece interfaces released into an indoor environment. The 30-min time-weighted-average aerosol concentration recorded was 0.072 ± 0.001 mg m^−3^ when using a jet nebuliser open-facemask combination. The effect of simulated aerosol treatment during high-flow therapy on fugitive emissions is unclear from the literature.

This study examines fugitive emissions during aerosol drug delivery in combination with high- flow therapy. Fugitive emissions are a potential source of secondary inhalation exposure from medical aerosols that may occur in clinical settings. A number of key factors were examined that could influence secondary exposure, including supplemental gas-flow rate, patient type and distance from the interface.

## 2. Materials and Methods

### 2.1. HFNT Circuit and Interfaces

The Optiflow system (Airvo 2, Fisher & Paykel Healthcare, Auckland, New Zealand) was used. An adult breathing circuit (P/N: 900PT552) was used with an adult nasal cannula (P/N: OPT 944) or a tracheostomy interface (P/N OPT 970) with a tracheostomy tube (Shiley Inner Cannula Tracheostomy Tube ID 6.4mm, Medtronic, Galway, Ireland). A paediatric breathing circuit (P/N: 900PT531) was used with a paediatric nasal cannula (P/N: OPT 318) or a tracheostomy interface (P/N OPT 970) with a tracheostomy tube (Shiley Paediatric Tracheostomy Tube ID 5.5mm, Medtronic, Galway, Ireland). The Airvo 2 HFNT system features a humidifier with an integrated flow source and was used with a nebuliser adapter. The nebuliser adapter was positioned at the humidification chamber. All testing was completed at clinically relevant gas-flow rates (10 L/min, 40 L/min and 60 L/min for simulated adult HFNT and 2 L/min, 10 L/min and 20 L/min during simulated paediatric HFNT). A tracheostomy interface is not operated at 2 L/min in the clinical setting, as a result, no runs were performed at this setting.

### 2.2. Inhaled Dose

Nasal cannulas were positioned in the nose of anatomically relevant nose–throat models, in accordance with manufacturers’ instructions. The nose–throat models were connected to a breathing simulator (Harvard Apparatus Dual Phase Control Respirator, Harvard Apparatus, MA, USA) via a collecting filter (RespirGard II 303, Baxter, Dublin, Ireland). A healthy adult breath pattern (tidal volume 500 mL, breath rate 15 BPM and inspiratory: expiratory ratio 1:1) and a healthy paediatric breathing pattern (tidal volume 250 mL, breath rate 25 BPM and inspiratory: expiratory ratio 1:2) were used. Inhaled dose was determined by quantifying the mass of drug on a filter positioned distal to the trachea. The tracheostomy interface and tracheostomy tube were attached directly to a collecting filter. Additional figures containing photographs of the three different combinations of the test rigs are supplied as supplementary material (Figures S1–S3).

The humidifier was powered on and allowed to come to temperature (37 degrees Celsius) and a 2 mL dose of albuterol sulphate (2 mg/mL) (GlaxoSmithKline Ltd., Dublin, Ireland) was nebulised. Experiments were performed using the Aerogen Solo, vibrating mesh nebuliser (Aerogen Ltd., Galway, Ireland). Albuterol was used as it is a commonly nebulised drug utilised in the characterisation of aerosol drug delivery systems. At the end of each dose administration, the drug captured on a filter was eluted using 10 mL of deionised water. The mass of drug was quantified by means of UV spectrophotometry at a wavelength of 276 nm and interpolation on a standard curve of albuterol sulphate concentrations (200 to 3.125 µg/mL). Results for inhaled dose are expressed as the percentage of the nominal dose placed in the nebuliser’s medication cup.

### 2.3. Anatomical Models

A previously described, an airway model of the adult nose–throat region was used as the adult model [29]. A model of a small child nose–throat region (nasal cavity, pharynx and larynx) with a volume of ~22.3 cm^3^ is based on a scan of a 5-year-old female, and was used as the paediatric model [30]).

### 2.4. Characterising Fugitive Emissions

**Figure 1:**
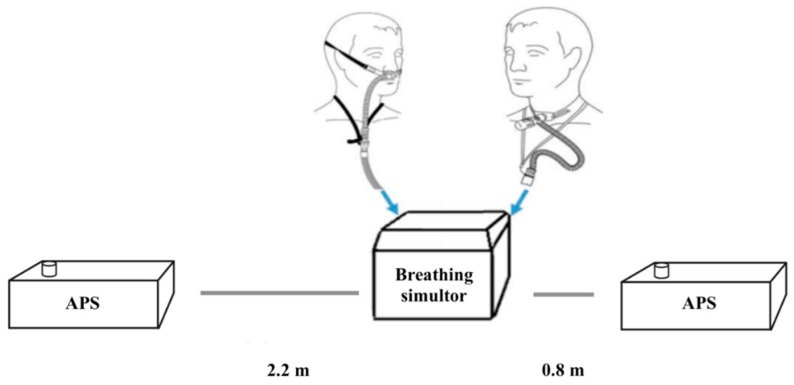
APS at varying distances relative to the simulated patient (ASL 5000) with nasal cannula interfaces.

The aerodynamic particle sizer (APS) (APS, model 3321 TSI Inc., St. Paul, MN, USA) was the primary aerosol monitoring instrument used in this study. Throughout the experiments, the APSs continuously measured aerosol mass concentrations and size distributions (0.5 to 20 μm) of the airborne concentration in the room. The APSs were located at a distance of 0.8 m and 2.2 m away from the patient interface (Figure 1). The two distances were chosen on the basis that the distance of 0.8 m was approximately one arm’s length away, simulating a caregiver holding a nebuliser/facemask to a patient’s face. The distance of 2.2 m was selected as it approximately represented the distance between beds in a primary care centre, simulating a patient situated in a bed next to the patient receiving the aerosol therapy.

The APSs were set to record data at 20 s intervals for a total of 30 min. The initial five-minute period established a baseline measurement of ambient aerosol in the room prior to activating the nebuliser. The remaining 25 min was selected to monitor for fugitive emissions during the nebulisation period and the consequent aerosol decay. After each nebulisation event, the laboratory was vented, and the aerosol concentration was monitored until it returned to ambient levels.

### 2.5. Temperature, Humidity and Airflow Characteristics

The laboratory room in which the study was conducted had dimensions L = 6.06 m, W = 2.70 m and H = 2.71 m. The air change rate was measured using the tracer gas decay method with CO2 as the tracer [31], and a GrayWolf probe IQ-610 (GrayWolf Sensing Solutions; Shelton, CT, USA) was used for gas detection. The air change rate was calculated to be approximately 0.65 h^−1^.

Room temperature and relative humidity in the laboratory room were measured using a digital hygro-thermometer-datalogger (Model DHM200, Pacer Instrument, Chippewa Falls, WI, USA) and values recorded were in the ranges 18.5 to 23.8 °C and 47.3 to 66.9%, respectively. The experiments took place over a three-week period.

### 2.6. Data Analysis and Statistics

The data analysis for this study was performed using statistical package IBM SPSS Statistics 24 (IBM Corp., Armonk, NY, USA, 2013). Summary and descriptive statistics were performed on the aerosol concentrations. All distribution characteristics are summarised by arithmetic mean and standard deviations.

## 3. Results

### 3.1. Inhaled Dose

The overall trend, as shown in Table 1, indicated that for both the nasal cannula and tracheostomy interfaces, as the flow rate increased, there was a corresponding decrease in inhaled dose (%).

When the adult scenarios were compared under the same flow-rate conditions, similar inhaled doses were found between the nasal cannula and the tracheostomy interface. However, there was a stark contrast when the paediatric scenarios were compared. The paediatric nasal cannula only delivered 2.5% to 11% of the tracheostomy dose under the same flow-rate conditions. The paediatric nasal cannula scenarios had a lower inhaled dose than the adult scenarios, while the paediatric tracheostomy scenarios had a higher inhaled dose than the adult scenarios.

When comparing the inhaled dose for the tracheostomy interface and the nasal cannula for the paediatric and adult patients under the same flow-rate conditions (10 L/min), there was a noticeable difference in the inhaled dose, which indicated that additional factors were influencing drug delivery.

### 3.2. Peak Aerosol Concentrations

**Figure 2:**
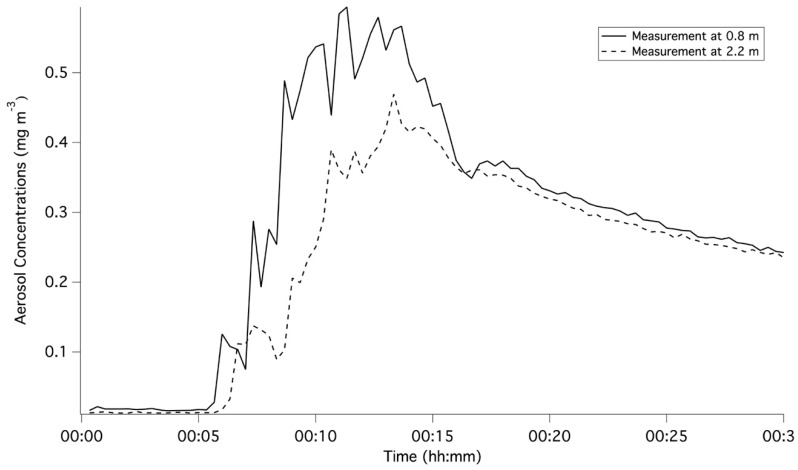
Comparison of time-series averaged aerosol concentrations for the three runs with a gas-flow rate of 10 L/min at two distances using the tracheostomy interface for a simulated adult.

**Figure 3:**
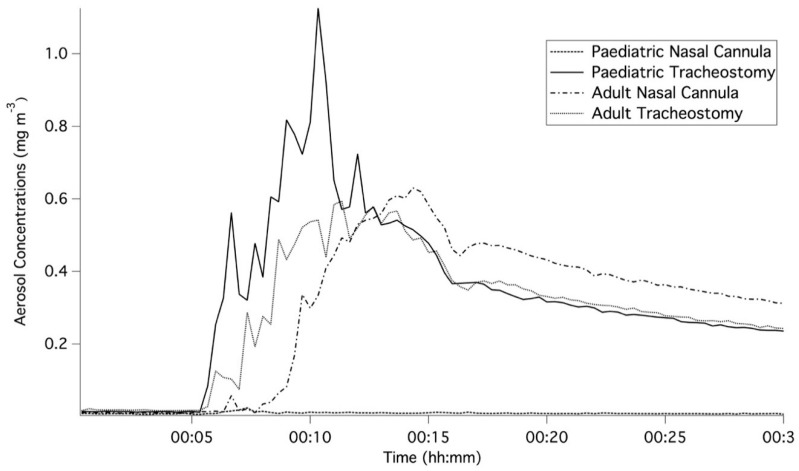
Comparison of the time-series averaged aerosol concentrations for the three runs recorded for all interfaces with a gas-flow rate of 10 L/min and at a distance of 0.8 m from the setup.

Figure 2 and Figure 3 represent the averaged for each of the three runs per scenario and demonstrate the initial five-minute baseline of ambient concentration, the increase in aerosol concentration during the nebulisation period and the consequent decay following the nebulisation. The increase in aerosol concentrations can be attributed to fugitive emissions from the high-flow therapy interfaces. Table 2 summarises the peak aerosol concentrations recorded at a distance of 0.8 m from the patient interface. Across all scenarios, the ratio of averaged peak measurements at respective distances of 2.2 and 0.8 m ranged from 0.20 to 1.27. As shown in Figure 2, the main differences arose during the period between five and fifteen minutes after the commencement of nebulisation. During this period, proximity to the interface resulted in higher concentrations. However, once the nebulisation ceased, concentrations at the two distances converged and were comparable for the remaining 15 min.

The peak concentration was reached between 7 to 15 min for the distance of 0.8 m away from the patient interface, and between 9 to 15 min for the 2.2 m distance. However, the majority of the peak concentrations occurred between 10 and 14 min corresponding to the duration of the nebulisation period, highlighting the cumulative fugitive emissions from the high-flow therapy interfaces.

### 3.3. Time-Weighted Averaged Aerosol Concentrations

The mean and standard deviation of the averaged five-minute baseline ambient aerosol concentration was 0.011 ± 0.008 mg m^−3^. Table 3 summarises the 30-min time-weighted averages for the nasal cannula and tracheostomy scenarios for adult and paediatric patients at a distance of 0.8 m and 2.2 m. For all scenarios where the air-flow rate was below 20 L/min, higher aerosol concentrations were recorded at a distance of 0.8 m from the patient interface. However, when the air-flow rate was 40 L/min or above, higher concentrations were recorded at a distance of 2.2 m due to the increasing dispersion of the aerosol throughout the room. For both the nasal cannula and the tracheostomy during simulated adult breathing, there was a decrease in aerosol concentrations with increasing gas-flow rate. While both interfaces yielded comparable aerosol concentrations at 10 L/min, there was a greater decrease in concentration for the nasal cannula in comparison with the tracheostomy at higher flow rates. At 60 L/min, the tracheostomy aerosol concentrations were twice those of the nasal cannula concentrations.

Different trends were observed for the nasal cannula and tracheostomy during paediatric breathing. The highest aerosol concentration was observed at the lowest gas-flow rate for the nasal cannula, while the highest concentration was observed at the highest gas-flow rate for the tracheostomy interface.

There was a noticeable difference between the aerosol concentrations measured for the nasal cannula and the tracheostomy interface during simulated paediatric breathing. However, the same variations did not occur between the nasal cannula and tracheostomy interface for the adult patient or between adult and paediatric patients with the tracheostomy interface.

### 3.4. Aerosol Droplet Sizing

Table 4 summarises the average mass median aerodynamic diameter (MMAD) over the 30-min period, in terms of mass concentrations recorded by the APS at a distance of 0.8 m away from the patient interface.

When considered in combination with Table 3, the MMAD for the paediatric breathing patterns for the nasal cannula interface are reflective of the ambient aerosol concentrations, not the fugitive emissions released from the nebuliser system. For the remaining combinations, all MMADs were in 1 to 2 µm for both the 0.8 and 2.2 m distances.

The MMAD was comparable for the tracheostomy interface and the nasal cannula at 10 L/min, regardless of the patient interface or gas-flow rates. Overall, as the gas-flow rates increased, the MMAD across all patient interfaces decreased.

### 3.5. Potential Inhalation Exposure

As a worked example, two different types of patient interfaces for high-flow therapy were examined, during aerosol delivery, to estimate the potential inhalation exposure. The first was a caregiver, who was assumed to be at the distance of 0.8 m from the patient, while the bystander was assumed to be at the distance of 2.2 m. Inhalation exposure was calculated for the adult nasal cannula scenario and the paediatric tracheostomy, both at 10 L/min, using the time-series averaged data for each scenario. To focus solely on the fugitive emissions from each interface, the five-minute baseline average of the aerosol concentration was subtracted from the time-series data. Short-term exposure inhalation rate of 1.3 × 10^−2^ m^3^/min and 4.8 × 10^−3^ m^3^/min were selected to represent the caregiver and the bystander, respectively. The caregiver was assumed to have a ‘light intensity’ activity level, while the bystander was assumed to have a ‘sedentary/passive’ activity level [32]. In this scenario, the caregiver and the bystander were estimated to be potentially exposed to 8.5% and 3.2% of the original drug, respectively, for the nasal cannula interface, with corresponding values of 10.2% and 2.3% for the tracheostomy interface, respectively.

## 4. Discussion

This study examined the potential for secondary inhalation of medical aerosols emitted during high-flow therapy. Fugitive emissions were examined for both a nasal cannula and a tracheostomy interface under three different gas-flow rates during simulated paediatric and adult breathing. For each patient, a breathing simulator and a head model with a nasal cannula or a tracheostomy interface with a tracheostomy tube, were used to simulate healthy adult and paediatric breathing. Once released into the environment, the APS characterised time-series aerosol concentrations and size distributions, which were reflective of the fugitive emissions from high-flow interface selection. While the current methodology did not isolate the fugitive emissions from the ambient aerosol, the changes in time-series concentrations highlighted that ambient aerosol was only a minor contributor as shown in Figure 2 and Figure 3. The increases in aerosol concentrations were attributed to the release of fugitive aerosol into the surroundings.

The current approach did not differentiate between aerosol that was released prior to inhalation, or aerosol that was possibly exhaled after inhalation. While the use of collection filters has been widely used as a suitable method to determine the inhalation dose [33,34], it might not be truly representative of a simulation of real exhalation. However, since the overall percentage of the inhaled dose is relatively small, it is assumed that the majority of the fugitive emissions consists of aerosol escaping prior to inhalation.

Vibrating mesh nebuliser technology generates aerosol by a contraction and expansion of a vibrational element producing movement on an aperture plate to produce an aerosol; this approach does not require any compressed air to generate the aerosol. The size of the aerosol and flow are determined by the exit diameter of the aperture holes [35]. Previous research showed that the residual mass remaining in a VMN was independent of the patient interface, heating and humidification settings [26,28]. As the same VMN was used for each scenario, the aerosol generation rate should be consistent across all scenarios with changes in inhaled dose and fugitive emissions influenced by the patient interface, the gas-flow rate and the breathing profiles.

The results indicate that the inhaled dose (%) decreased with an increased gas-flow rate. The only exception was the nasal cannula at the paediatric breathing profiles at 10 L/min, which had a lower inhaled dose than at 20 L/min. Although there is no clear explanation for this result, one potential reason may be that at 20 L/min more aerosol was forced through the system, including, perhaps, some drug that has rained out in the head model. All the remaining trends were consistent with other studies. Perry et al. [13] examined the inhaled dose during high-flow nasal cannula therapy using the Aerogen Solo device. For simulated adult breathing, the inhaled doses were found to be 0.8% and 0.2% for gas-flow rates of 10 and 40 L/min, respectively. However, this study found 5.35 ± 2.81% and 2.56 ± 1.38% for 10 and 40 L/min, respectively. During simulated paediatric breathing, 1.2, 0.1 and 0% were found for gas-flow rates of 3, 10 and 20 L/min, as compared to this study’s findings of 3.23 ± 1.58, 0.42 ± 0.06 and 1.17 ± 0.28% for flow rates of 2, 10 and 20 L/min. Dailey et al. [36] examined the inhaled dose using a high-flow nasal cannula and reported considerably higher values of 26.7% to 27.4% at 10 L/min, 11.6% to 14.2% at 30 L/min and 3.5% to 5.9% at 50 L/min. Perry et al. [13] used a lower tidal volume for the paediatric breathing profiles setting than the present study; while Dailey et al. used an inspiratory-expiratory ratio of 1:2, an Albuterol sulphate mixture (2.5 mg/0.5 mL). Neither study reported the length of the tubing or the internal diameter of the nasal prongs making a direct comparison difficult. While there are differences between the studies at each flow rate, the overall trends remain similar. Direct comparisons between inhaled dose are difficult due to several factors influencing the drug available for inhalation, such as the position of components in the ventilator circuit, simulated breathing settings and humidification affect the inhaled dose [37,38,39]. Golshahi et al. [40] reported that increasing the tubing length from 20 cm to 50 cm increased the deposition fraction in the tube from 1.2% to 13.3%; this compared with a tube of length 1.8 m used in the current study.

The higher the inhaled dose, the higher the fugitive emissions. This indicated that the majority of the loss mechanisms occurred prior to exiting the patient interface. Dailey et al. [36] reported that increased flow rate could increase turbulence and impactive loss of aerosol within the tubing, which means less aerosol is reaching the patient interface and available for inhalation and/or to be released as fugitive aerosol.

In this study, a vibrating mesh nebuliser was used to generate the medical aerosol, previous work reported that with aerosol generated from VMN has a MMAD of 4 µm [28]. However, the observed averaged MMAD of the fugitive emissions ranged from 1.248 to 1.793 µm across all scenarios. The aerosol released into the environment was considerably smaller than originally generated by the nebuliser. The larger sized aerosol, in combination with the air flow, experiences greater inertia that can increase the rate of impaction and deposition of the larger sized aerosol within the system and upon the head model. The consequence of this is that the fugitively emitted aerosol is dominated by a lower sized fraction of aerosol, decreasing the MMAD. Bhashyam et al. [11] reported the volume median diameter of aerosol in a nasal cannula aerosol delivery system using a 3 L/min driving flow. The aerosol exiting the nebuliser was 5.0 ± 0.2 μm, which reduced to 2.2 ± 0.2 μm from an adult cannula and 1.9 ± 0.3 μm from a paediatric cannula. Although higher gas-flow rates were used in the current study, similar trends were found with a decrease in the size fraction and the size leaving the patient interface. Berlinski [34] reported that the aerosol size fraction exiting the tip of the tracheostomy tube was 1.20 to 1.77 µm. Berlinski [33] reported that aerosol changed its characteristics when travelling through a tracheostomy tube; the effects were more noticeable for nebulised aerosol than metered dose inhalers or valved aerosol reservoirs. Additionally, during simulated adult breathing with gas-flow rates of 10, 40 and 60 L/min, there were comparable trends between the nasal cannula and the tracheostomy interface. This could indicate that the size distributions of fugitive emissions are independent of the patient interface and are governed instead by the flow velocities in these scenarios. The size fraction of the released aerosol has implications, as the aerosol can remain airborne for longer periods, which has been linked to secondary inhalation exposure [41].

Aerosol concentration is governed by local factors including room dimensions and layout, air turbulence, air-flow rates and temperature that influence dispersion and decay [41,42,43]. However, previous research by McGrath at al. [44] was conducted in the same laboratory that allows for comparisons between the current study and fugitive emissions from a standard jet nebuliser using manufacturer-prescribed 8 L/min. Adult and paediatric head models were connected to patient interfaces; a facemask and mouthpiece without an expiratory filter on the exhaled port. When adjusted to reflect the analysis in the present study, aerosol concentration ranged from 0.221 to 0.323 mg m^−3^, which is comparable with the observed concentration range of 0.290 to 0.370 mg m^−3^ in the current study.

While this study indicates that the caregiver is exposed to higher fugitive emissions than a bystander, due to proximity to the patient interface, it is worth noting the calculated exposure ratios for bystanders versus caregivers. The caregiver inhaled 8.5% of the original drug using the nasal cannula, as compared to 10.2% for the tracheostomy. However, the bystander inhaled 3.2% for the nasal cannula scenario compared with 2.3% in the tracheostomy scenario. This was due to occurrence of the peak exposure, which has been previously documented in indoor air quality studies [45,46].

There was a contrast between the aerosol concentrations recorded during the nasal cannula scenario for the paediatric and adult patients, which was not reflected in the tracheostomy scenarios. The paediatric nasal cannula had smaller tubing dimensions and components, which could have affected the aerosol exiting the interface. For the adult nasal cannula (OPT 944), the inner diameter of the nasal prongs was 3.5 mm at the narrowest point, while for the paediatric nasal cannula (OPT 318), the inner diameter of the nasal prongs was 2 mm at the narrowest point. Studies have reported that the characteristics of the paediatric setup reduced the aerosol exiting the cannula during high-flow therapies [13,47]. Since the same tubing and tracheostomy interface (12 mm at the narrowest point) was used for the adult and paediatric tracheostomy, with different breathing profiles, this could explain why the same differences were not observed in this case.

## 5. Conclusions

Fugitive emissions released during aerosol therapies present potential risks to healthcare workers in a clinical setting. To date, only limited studies exist that have examined fugitive emissions, once released into the surroundings. No studies have previously quantified time-series aerosol concentrations and the size distribution of fugitive emissions from high-flow therapy. Potential inhalation exposure to fugitive emissions during high-flow aerosol therapy was examined using adult and paediatric scenarios for both nasal cannula and tracheostomy interfaces. This study confirmed the release of fugitive emissions into the environment during aerosol high-flow treatment. The results showed that variations in interface type, patient (adult or paediatric) and gas-flow rates all impacted the quantity and characteristics of fugitive emissions. The highest aerosol concentration of 0.370 ± 0.046 mg m^−3^ was recorded from the paediatric tracheostomy with a gas-flow rate of 20 L/min, which also corresponded to the second highest inhaled dose across all the scenarios. It was observed that as the gas-flow rate increased, the inhaled dose decreased, and the fugitive emissions decreased. Overall, the results can be used to develop approaches for healthcare providers and patients to inform best practice to mitigate the risks of fugitive emissions in clinical practice.

## Figures and Tables

**Table 1:** Inhaled dose recorded during each nebulisation event using simulated adult and paediatric breathing profiles for the nasal cannula and tracheostomy interfaces, mean and standard deviations over the three events.

Breathing Pattern/Gas-Flow Rate	% Inhaled Dose	
	Nasal Cannula	Tracheostomy
Paediatric/2 L/min	3.23 ± 1.58	N/A
Paediatric/10 L/min	0.42 ± 0.06	16.77 ± 0.26
Paediatric/20 L/min	1.17 ± 0.28	10.65 ± 2.54
Adult/10 L/min	5.35 ± 2.81	6.39 ± 1.58
Adult/40 L/min	2.56 ± 1.38	1.49 ± 0.30
Adult/60 L/min	1.01 ± 0.26	1.36 ± 0.14

**Table 2:** Peak aerosol concentration recorded at a distance of 0.8 m from the patient interface. Each nebulisation event using simulated adult and paediatric breathing profiles for nasal cannula and tracheostomy interfaces, mean and standard deviations for each scenario.

Breathing Pattern/Gas-Flow Rate	Peak Aerosol Concentration (mg m^−3^)	
	Nasal Cannula	Tracheostomy
Paediatric/2 L/min	0.081 ± 0.016	N/A
Paediatric/10 L/min	0.025 ± 0.008	1.353 ± 0.212
Paediatric/20 L/min	0.017 ± 0.006	1.241 ± 0.160
Adult/10 L/min	0.636 ± 0.067	0.730 ± 0.073
Adult/40 L/min	0.196 ± 0.049	0.278 ± 0.013
Adult/60 L/min	0.090 ± 0.004	0.188 ± 0.039

**Table 3:** The 30-min averaged mass concentration at a distance of 0.8 m and 2.2 m for the adult and paediatric scenarios using the nasal cannula and tracheostomy interfaces, mean and standard deviations over the three events.

Breathing Pattern/Gas-Flow Rate	Average Mass Concentration (mg·m^−3^)			
	Measurements at the Distance of 0.8 m		Measurements at the Distance of 2.2 m	
	Nasal Cannula	Tracheostomy	Nasal Cannula	Tracheostomy
Paediatric/2 L/min	0.037 ± 0.005	N/A	0.027 ± 0.008	N/A
Paediatric/10 L/min	0.009 ± 0.001	0.334 ± 0.028	0.008 ± 0.002	0.211 ± 0.008
Paediatric/20 L/min	0.005 ± 0.001	0.370 ± 0.046	0.005 ± 0.002	0.210 ± 0.040
Adult/10 L/min	0.302 ± 0.027	0.290 ± 0.027	0.277 ± 0.050	0.236 ± 0.124
Adult/40 L/min	0.093 ± 0.006	0.138 ± 0.005	0.109 ± 0.026	0.176 ± 0.039
Adult/60 L/min	0.050 ± 0.003	0.101 ± 0.011	0.061 ± 0.042	0.127 ± 0.030

**Table 4:** Summary of the averaged mass median aerodynamic diameter during each nebulisation event when using two interfaces (nasal cannula and tracheostomy) with three gas-flow rates for simulated adult and paediatric patients, mean and standard deviations over the three events.

Breathing Pattern/Gas-Flow Rate	Average MMAD (µm)	
	Nasal Cannula	Tracheostomy
Paediatric/2 L/min	1.331 ± 0.043	N/A
Paediatric/10 L/min	2.070 ± 0.531	1.784 ± 0.033
Paediatric/20 L/min	3.200 ± 0.532	1.577 ± 0.134
Adult/10 L/min	1.722 ± 0.216	1.793 ± 0.111
Adult/40 L/min	1.259 ± 0.197	1.248 ± 0.131
Adult/60 L/min	1.350 ± 0.034	1.300 ± 0.023

## Supplementary Materials

The following are available online at https://www.mdpi.com/1999-4923/11/6/254/s1, Figure S1: A photograph highlighting the physical setup for the paediatric nasal cannula set up, Figure S2: A photograph highlighting the physical setup for the adult nasal cannula set up, Figure S3: A photograph highlighting the physical setup for the adult and paediatric tracheotomy set up.
