# Supplementary material for: Investigation of Fugitive Aerosols Released into the Environment during High-Flow Therapy
Source: Pharmaceutics. 2019 Jun 1;11(6):254. doi: 10.3390/pharmaceutics11060254 (PMC6630289; doi:10.3390/pharmaceutics11060254)
Supplement: Supplementary file 1 [file pharmaceutics-11-00254-s001.pdf]

pharmaceutics

Supplementary Materials: Investigation of Fugitive Aerosols Released into the Environment during High Flow Therapy

James A. McGrath, Ciarraí O'Toole, Gavin Bennett, Mary Joyce, Miriam A. Byrne and Ronan MacLoughlin

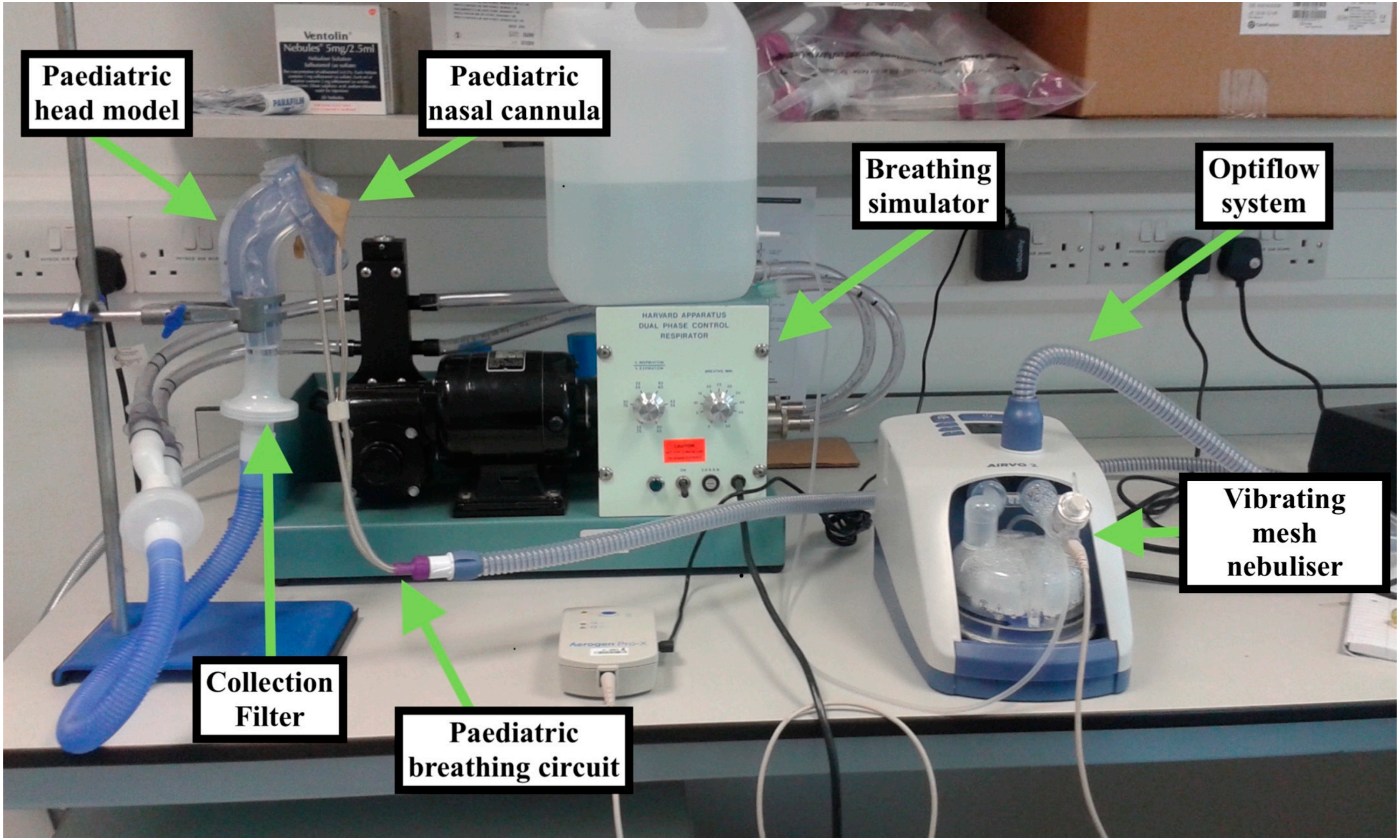

Figure S1. A photograph highlighting the physical setup for the paediatric nasal cannula set up.

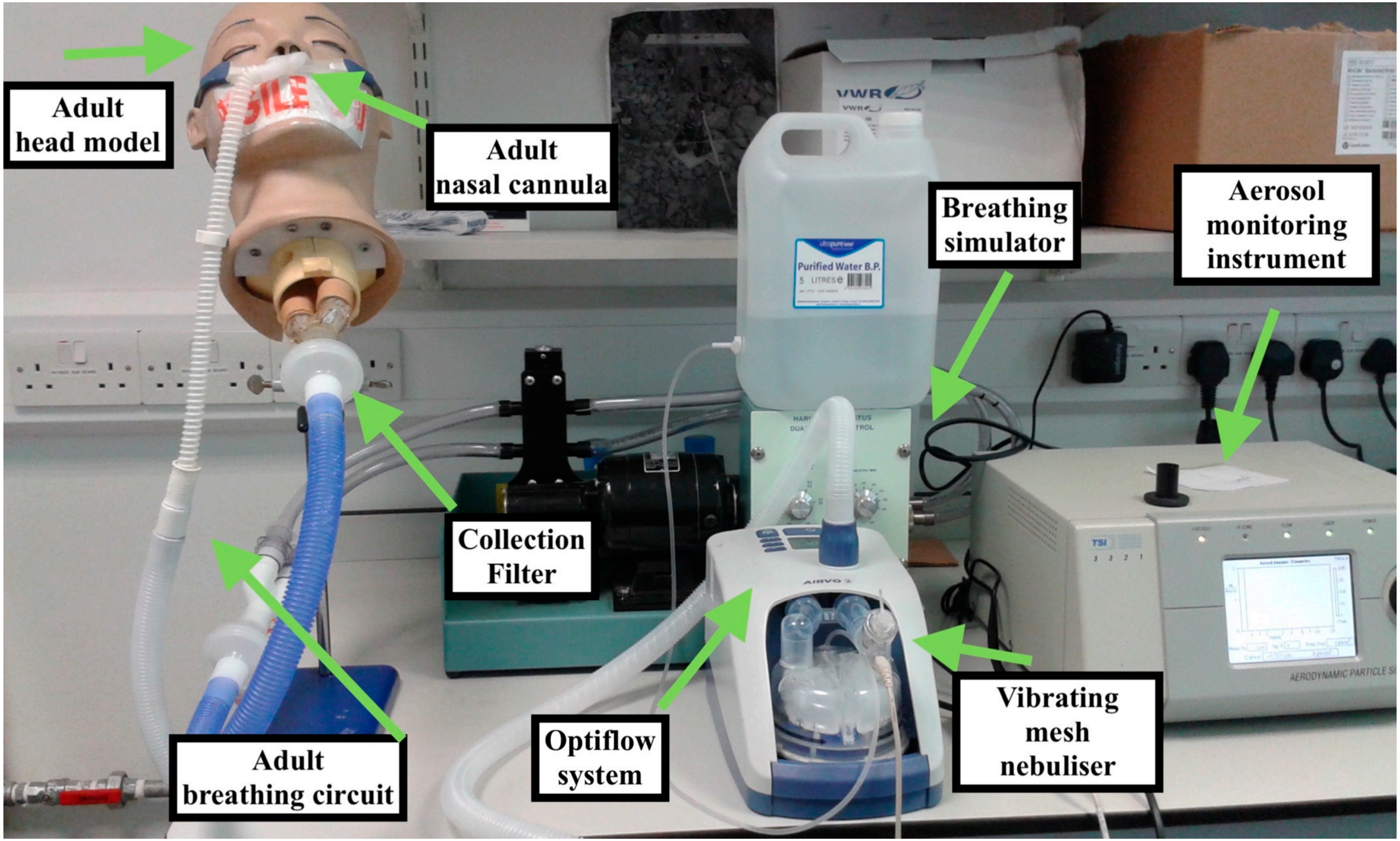

Figure S2. A photograph highlighting the physical setup for the adult nasal cannula set up.

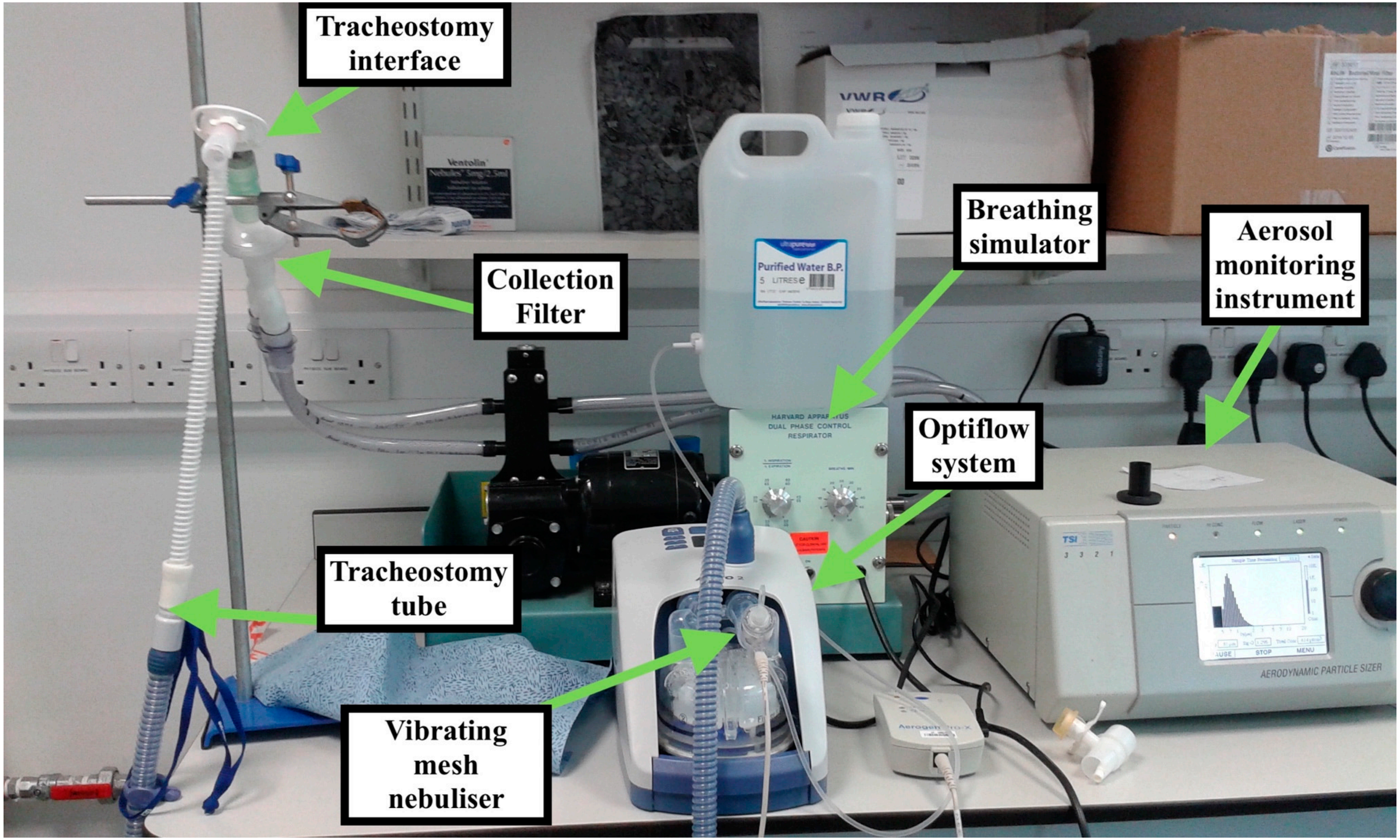

Figure S3. A photograph highlighting the physical setup for the adult and paediatric tracheotomy set up.
